# C-754-03. Enoxaparin Dosing in Burn Patients - Efficacy of a Weight-Based Dosing Protocol

**DOI:** 10.1093/jbcr/irag033.031

**Published:** 2026-04-06

**Authors:** Nolan Wilson, Ken Gorsegner, Uyen Dinh, Adis Keric, Alexandra M Lacey

**Affiliations:** Regions Hospital Burn Center, Minneapolis, MN; Regions Hospital, Saint Paul, MN; Regions Hospital, Saint Paul, MN; Regions Hospital, Saint Paul, MN; Regions Hospital, Saint Paul, MN

## Abstract

**Introduction:**

There are currently no standardized guidelines for enoxaparin dosing for venous thromboembolism (VTE) prophylaxis in burn patients. Previous research indicates that the common 30 mg twice daily dosing is inadequate for burn patients and leads to sub-therapeutic anti-factor Xa levels in up to 79% of patients. In 2017, our institution instituted weight-based dosing for burn patients of enoxaparin 0.5 mg/kg every 12 hours or 0.4 mg/kg every 12 hours for BMI 40 or greater. The purpose of this quality improvement project was to evaluate the efficacy of our protocol by determining the number of patients that reached goal anti-Xa level of 0.2-0.4 IU/mL with initial anti-Xa check based on dosing per protocol.

**Methods:**

This was a retrospective, single-center quality improvement project. Before 2017, burn patients at our institution received enoxaparin 30 mg every 12 hours or 40 mg every 12 hours for BMI ≥35. This dosing protocol was defined as pre-protocol dosing. After 2017, patients received enoxaparin 0.5 mg/kg every 12 hours if BMI < 40 and 0.4 mg/kg every 12 hours if BMI ≥40 with a maximum initial dose of 60 mg twice daily. This dosing protocol was defined as post-protocol dosing. Only adult patients were included in this study.

The final sample was split into two groups for comparison: pre-protocol and post-protocol patients. Patient characteristics included age, BMI, CrCl, burn TBSA, and diagnosis. A sample of 133 patients was determined to be adequately powered (80% power, 2-sided alpha 0.05) to detect a 25% difference in reaching goal at first anti-factor Xa check. Students t-tests and χ^2^ tests were used to analyze continuous and categorical data, respectively. A *p*-value of <0.05 was considered statistically significant.

**Results:**

33.3% of pre-protocol patients and 59.6% of post-protocol patients reached goal anti-Xa at initial check (*p*=.004). Average age, BMI and creatinine clearance were not significantly different between the two groups. Average burn TBSA was 24% for pre protocol and 11% for post protocol which was statistically significant.

**Conclusions:**

A significantly greater proportion of patients reached goal anti-Xa in the post-protocol group compared to the pre-protocol group. Future areas of study could include exploring occurrences of VTE in burn patient populations with various dosing strategies to determine if reaching goal anti-factor Xa more quickly has a clinical impact on reducing rate of VTE. Additionally, examining the number of bleeding events in burn patients on enoxaparin for prophylaxis would help determine the overall safety profile of more aggressive dosing protocols.

**Applicability of Research to Practice:**

Weight-based dosing of enoxaparin in burn patients results in higher occurrence of achieving goal anti-Xa levels for VTE prophylaxis.

**Funding for the study:**

N/A.

